# Estimation of Ethiopia’s immunization coverage – 20 years of discrepancies

**DOI:** 10.1186/s12913-021-06568-0

**Published:** 2021-09-13

**Authors:** Bob Pond, Abebe Bekele, Sandra Mounier-Jack, Habtamu Teklie, Theodros Getachew

**Affiliations:** 1 Independent public health analyst, Camas, WA 98607 USA; 2 Ethiopian Public Health Institute (EPHI), Addis Ababa, Ethiopia; 3 London School of Hygiene and Tropical Medicine (LSHTM), London, UK

**Keywords:** Immunization coverage, Data quality, Household surveys, Routine health information, Ethiopia

## Abstract

**Background:**

Coverage with the third dose of diphtheria-pertussis-tetanus-containing vaccine (DPT3) is a widely used measure of the performance of routine immunization systems. Since 2015, data reported by Ethiopia’s health facilities have suggested DPT3 coverage to be greater than 95%. Yet, Demographic and Health Surveys in 2016 and 2019 found DPT3 coverage to be 53 and 61% respectively for years during this period. This case study reviews the last 20 years of administrative (based on facility data), survey and United Nations (UN) estimates of Ethiopia’s nationwide immunization coverage to document long-standing discrepancies in these statistics.

**Methods:**

Published estimates were compiled of Ethiopia’s nationwide DPT3 coverage from 1999 to 2018. These estimates come from the Joint Reporting Form submitted annually to WHO and UNICEF, a series of 8 population-based surveys and the annual reports of the WHO/UNICEF Estimates of National Immunization Coverage (WUENIC). Possible reasons for variation in survey findings were explored through secondary analysis of data from the 2012 immunization coverage survey. In addition, selected health officials involved with management of the immunization program were interviewed to obtain their perspectives on the reliability of various methods for estimation of immunization coverage.

**Findings:**

Comparison of Ethiopia’s estimates for the same year from different sources shows major and persistent discrepancies between administrative, survey and WUENIC estimates. Moreover, the estimates from each of these sources have repeatedly shown erratic year-to-year fluctuations. Those who were interviewed expressed scepticism of Demographic and Health Survey (DHS) statistics. Officials of the national immunization programme have repeatedly shown a tendency to overlook all survey statistics when reporting on programme performance.

**Conclusions:**

The present case study raises important questions, not only about the estimation methods of national and UN agencies, but about the reliability and comparability of widely trusted coverage surveys. Ethiopia provides an important example of a country where no data source provides a truly robust “gold standard” for estimation of immunization coverage. It is essential to identify and address the reasons for these discrepancies and arrive at a consensus on how to improve the reliability and acceptability of each data source and how best to “triangulate” between them.

**Supplementary Information:**

The online version contains supplementary material available at 10.1186/s12913-021-06568-0.

## Background

Reliable monitoring of performance has been key to the success of immunization programs. Ethiopia, Africa’s second most populous nation, provides an important case study of the challenges of reliably estimating immunization coverage. Such estimation is undertaken annually by both the Federal Ministry of Health (FMoH) of Ethiopia and by the World Health Organization/UNICEF.

Ethiopia’s Expanded Programme for Immunization (EPI) aims to administer 11 different antigens to the more than 3 million infants born in the country each year. Vaccines are administered routinely at almost 20,000 health facilities throughout the country. “Static” (at the facility) delivery is supplemented with “out-reach” sessions in the community and large-scale periodic campaigns providing polio, measles and meningitis vaccine.

Coverage estimates have come from Demographic and Health Surveys (DHS [1]), typically repeated each 5 years, and special surveys focussed exclusively on immunization coverage [2] (hereafter referred to as Expanded Program for Immunization surveys or “EPI surveys”). In Ethiopia, the EPI Surveys of 2001 and 2007 employed non-probability sampling which had the potential to introduce non-sampling error [3]. In contrast, the EPI survey of 2012 used strict probability sampling. The most reliable information on immunizations given to a child comes from data recorded on a home-based record (HBR; sometimes supplemented with data from immunization registers kept at a nearby health facility) that is reviewed during the survey visit. Where an HBR is not available, the immunization status of the child is assessed through a series of questions posed to the caretaker. Such an assessment is said to be based upon “recall”.

As is done for other countries, the National Immunization Program (NIP) of Ethiopia uses data from their national Health Management Information System (HMIS) to calculate the annual “administrative estimate” of immunization coverage by dividing the reported annual total number of doses of a particular vaccine administered to infants (the numerator) by the official estimate of the population under 1 year of age (the denominator). As for most countries, Ethiopia’s NIP each year submits to WHO and UNICEF the Joint Reporting Form (JRF) [4, 5] including data on the number of doses administered and the official estimate of the denominator.

Since 2001, a panel of experts from these two UN organizations have met annually to reach a consensus known as the WHO/UNICEF Estimate of National Immunization Coverage or “WUENIC” [6, 7] (archived reports for 2001 to 2012 obtained by personal communication from Anthony Burton, WHO [8]). WUENIC estimates are not a separate data source but rather an interpretation of how best to reconcile multiple years of administrative estimates, survey estimates and other relevant information including reports from assessments of the HMIS system.

## Methods

### Secondary data analysis

This case study reviews estimates compiled from JRF and WUENIC reports published between 2000 and 2019 [5, 7, 8] of Ethiopia’s coverage with the third dose of diphtheria/tetanus/pertussis-containing vaccine (“DPT3”) which is widely used as a proxy for monitoring infant immunization activities. To understand the survey methods used, reports were also reviewed from 8 population-based surveys: the Ethiopia DHS’s of 2000, 2006, 2011, 2016 and 2019 [1], and three EPI surveys -- the 2001 EPI Survey (data summarized in each annual WUENIC report [7]), the 2006 EPI Survey [9] and the 2012 EPI Survey [10].

To explore possible reasons for discrepancies in survey findings, secondary analysis was performed with data from the 2012 EPI survey. For 595 (16%) of the 3762 children sampled for the 2012 EPI survey, data were obtained from both health facility immunization registers as well as caretaker recall. Comparison of the data for the same children from these two different sources permitted assessment of recall bias.

### Qualitative method

EPI focal persons at national, regional and zonal levels were interviewed from April to October 2017 to obtain their perspectives on the reliability of various methods for estimation of immunization coverage. Ethical clearance was obtained from the Institutional Review Board of Scientific and Ethical Review Office of EPHI. An information letter was addressed by EPHI to regional health bureaus, and zonal health offices for cooperation. Informed consent was obtained from all interviewees.

A total of 106 individuals were interviewed: one from each regional health team and one from each zonal health team -- with the exception of Hareri and Dire Dawa regions, where zonal health officials could not be reached. An open-ended standard questionnaire was administered face to face by researchers (see Additional file 1), focussing on the comparative reliability of methods for estimation of coverage and suggestions to improve the quality of estimates. Verbatim interviews were anonymised, transferred into Microsoft Excel and open text was analysed thematically. Themes included “reliability and confidence in data collection methods”, “strengths and weaknesses of methodologies” and “recommendations for improving the reliability and accuracy of individual data collection method”.

## Results

### Summary of the estimates

**Fig. 1:**
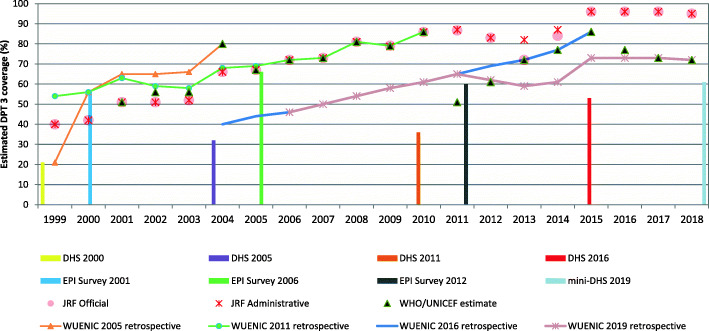
Summary of trends in estimates of Ethiopia’s DPT3 coverage among children 12 to 23 months of age, 1999 to 2018 – administrative, official, WHO/UNICEF contemporaneous (black triangles represent the first-ever WUENIC estimate for the cohort of infants born in that year), survey and WHO/UNICEF retrospective from select years

Figure 1 summarizes administrative, survey and WUENIC estimates of Ethiopia’s nationwide DPT3 coverage among children 12 to 23 months of age from 1999 to 2018. The Figure includes two different types of WUENIC estimates – (i) values that were published for each year (the black triangles represent the first “WHO/UNICEF estimate” ever published for the cohort of infants born in that year – e.g. the estimate for the 2006 birth cohort was published in 2007, but shown for 2006) and (ii) several retrospective WUENIC interpretations of the trend. Note that as new evidence becomes available each year, the WUENIC team may update its retrospective interpretation of the trend in coverage. To keep the chart as simple as possible, Fig. 1 shows only the retrospective trend lines published in 2005, 2011, 2016 and 2019.

We can understand this complex picture as follows:

1. Based upon findings from the 2001 EPI survey (the vertical blue bar), WUENIC estimators assumed from 2001 until 2005 that administrative data *under*-estimated true coverage by 14 percentage points (see the revisions for 2003 to 2005 [8]). This is implied by WUENIC’s retrospective trend analysis published in 2005 (the orange line).
2. The WHO/UNICEF panel as well as officials with the FMoH were surprised when results of the 2005 DHS later became available suggesting DPT3 coverage of only 32% in 2004 (the vertical purple bar). To verify the coverage, an EPI Survey was conducted in 2006. This survey suggested that DPT3 coverage was 66% in 2005 (the vertical green bar) when the administrative estimate was 67%. The WHO/UNICEF panel then retrospectively concluded that Ethiopia’s administrative estimates were now reliable. Henceforth, until the next coverage survey was conducted in 2011, after a six-year gap, true coverage was assumed to be equal to a steadily climbing administrative estimate (as shown by the green retrospective analysis of 2011). Review of financial data available on the website of the Global Alliance for Vaccines and Immunizations shows that, during this six-year period, the Alliance awarded $13.96 million of “ISS rewards” for the steady increases in the number of children reported to have been given their 3rd dose of DPT [11, 12].
3. The WUENIC panel and the FMoH were unpleasantly surprised when results of the 2011 DHS became available suggesting DPT3 coverage of only 36% in 2010 (the vertical orange bar). To verify the coverage, an EPI Survey was conducted in 2012 suggesting that DPT3 coverage was 60% in 2011 (the vertical dark grey bar) when the administrative estimate was 87%.
4. In 2014, in-country WHO and UNICEF staff reached consensus with the national immunization program (NIP) regarding immunization estimates (refer to the 2013 description of 2013 revision of the WUENIC report [8]). The NIP released an official estimate of 2013 DPT3 coverage (72% -- the pink circle for 2013) that was 10 percentage points lower than the administrative estimate (82% -- the red asterisk for 2013) and WUENIC endorsed this lower estimate (the black triangle of 72% for 2013). The WUENIC estimates for 2014 and 2015 were likewise based on the assumption that true coverage was 10 percentage points lower than the administrative estimate – even as this climbed to 96% for 2015.
5. The WUENIC panel and the Ministry were once again unpleasantly surprised when results became available from the 2016 DHS (the vertical red bar; DPT3 = 53%). For their most recent (2019 revision) estimate, the WUENIC Team did not use the findings from the 2019 mini-DHS (vertical  pale blue bar; DPT3 = 61%) because the survey report did not provide information to compute a recall bias adjustment factor as was done for previous surveys. Meanwhile, the FMoH are arranging for another independent EPI Survey to be conducted in 2021, accompanied with collection of blood samples for assessing sero-prevalence of protective anti-bodies against tetanus (Personal communication, Abebe Bekele, EPHI)

Review of Ethiopia’s official estimates of the number of surviving infants [5] suggests that fluctuation in the denominator account for little of the erratic fluctuations seen in administrative estimates (see Additional file 2).

Table 1 summarizes information about coverage surveys conducted in Ethiopia since 2000.

**Table 1:** Population-based surveys measuring national immunization coverage among children 12–23 months of age, Ethiopia, 2000–2019

Survey pair/ name	Year assessed	Sample size	Standard error	Data from documents^**a**^	Survey DPT3	Admin DPT3
Pair 1/2000 DHS	1999	2143	1.3%	27%	21%	40%
Pair 1/2001 EPI Survey	2000	3564	Not reported	52%	56%	42%
Pair 2/2005 DHS	2004	1877	1.9%	37%	32%	66%
Pair 2/2006 EPI Survey	2005	6903	Not reported	60%^b^	66%	69%
Pair 3/2011 DHS	2010	1927	1.9%	29%	36%	86%
Pair 3/2012 EPI Survey	2011	3762	2.5%	63%^b^	60%	87%
2016 DHS	2015	2004	2.2%	57%^b^	53%	96%
2019 mini-DHS	2017	1028	Not yet reported	Not yet reported	61%	96%

^a^Percentage of children surveyed for whom vaccination status was documented with either a home-based record or the facility immunization register

^b^For the EPI Surveys of 2006 and 2012 and for the DHS surveys of 2016 and 2019, data were transcribed from either HBRs or immunization registers kept at the nearest health facility

Each of the five DHS’s conducted in Ethiopia over the last 20 years derived a low percentage of their data from documents. Each of the first three DHS surveys was paired within a year with an EPI Survey with a larger sample size and a substantially higher percentage of immunization data based upon written documentation. For each of these three pairs, the EPI Survey estimated much higher levels of immunization coverage. Review of Fig. 1 shows that the WUENIC estimates for the years immediately following each of the three pairs of surveys reconciled administrative estimates with the result of the respective EPI survey while discounting the result of the respective DHS survey.

The EPI Surveys of 2001 and 2006 employed the non-probability sampling and data collection methodology previously specified by WHO for EPI cluster surveys [9, 13]. However, the EPI Survey of 2012 used strict probability sampling (e.g. mapping and listing of households followed by simple random sampling), applied rigorous data collection methods and took appropriate measures to assure independence and strong technical support and supervision [10].

Secondary analysis of data from the 2012 EPI survey was used to assess the direction and magnitude of recall bias. For 595 of the children surveyed, immunization records could be located at the nearest health facility even though an HBR was not available. Among these children, the DPT3 coverage as documented by facility records was 77.6% while the coverage of the same children based upon recall was 47.4%. Thus, recall resulted in major *under*-estimation of coverage, at least for this subset of children.

### The perspectives of national and sub-national health officials – qualitative findings

Interviewees varied in their perceptions of the strengths and weaknesses of the different methods for estimation of immunization coverage. Most health officials regarded the HMIS data as being more representative and comprehensive. *“With its limitations, HMIS is better since it reaches to the lowest administrative unit. HMIS depends on a continuous data collection and reporting system … it seeks information from the very periphery of the kebele”* (respondent from region #5).

However, respondents recognised factors limiting the quality of administrative estimates -- inadequate staff training, uncertain denominators and “false [over] reporting” (noted by informants in all regions) spurred by a desire to obtain recognition and promotion. “*In HMIS, if you report with high performance, you will be recognized and get advantage. So, this type of competition among workers leads to false report*” (respondent from region #1).

Most informants expressed scepticism about the reliability of DHS surveys and a lack of confidence in the sampling strategy and methodology, including the use of samples perceived as too small and unrepresentative; recall bias; insufficiently trained and supervised data collectors; and language barriers in some regions. *“EDHS is less reliable because the data quality and supervision is not as strong as coverage survey and HMIS. The result may be good or bad depending on the investigator, supervisor and data collectors.” (respondent from region #4).*

The long interval between DHS’s was seen as precluding timely availability of estimates. Nevertheless, a minority of interviewees recognised some strengths of the DHS including rigorous sampling and independence from “political pressure”. Most informants, however, felt that the EPI coverage surveys provided a better approximation of the true coverage, both because of the national ownership of the survey process and because the survey findings were seen by some informants as validating estimates based upon administrative data.. “*EPI coverage survey looks like reliable as the data shows the medium between EDHS and HMIS report. Other are not reliable. Maybe I don’t know the data collection process of EPI coverage survey but the report seems like an average one*.” (respondent from region #5).

Suggestions for improving the reliability of administrative estimates included investments in training and mentoring, regular supervision and better linkage with local communities. Disincentivising competition between Woredas was also mentioned as a means to encourage more truthful reporting. Some informants also recognized a need for more frequent surveys and more reliable estimates of the target population through enumeration at local level. Finally, a key recommendation was to routinely validate monthly reports with data from facility registers and the number of doses of vaccine supplied. “*Using electronic HMIS in all health facilities; visiting the health facilities and verifying the raw data in the registration; and comparing the registered data with tally and reported one in sampled facilities*.” (respondent from region #1).*“.*

The reluctance of NIP officials to accept survey statistics is reflected in the fact that the immunization sections of each of the last six Annual Performance Reports of the FMoH [14] have cited only administrative estimates – no survey estimates of immunization coverage have been included.

## Discussion

The reliability of administrative estimates is limited by errors in the reported number of doses administered as well as by unreliable estimates of the denominator [3, 15–18]. Too few doses are counted when reporting is not complete. Too many doses are counted due to either inadvertent double counting or intentional over-reporting. A meta-analysis of data quality audits commissioned by Gavi between 2001 and 2005 found that 20 of 47 countries, including Ethiopia, had “verification factors” of 0.80 or less, meaning that 20% or more of reported DPT3 doses could not be substantiated by records found at a health facility [19]. WUENIC reports for Ethiopia have also noted such evidence of over-reporting (for example, the descriptions for 2013 and 2014 imply verification factors of 0.88 and 0.97 respectively [8]). Unreliable estimates of the denominator can result from inaccurate counting of the total population during a population census, unreliable estimation of the crude birth rate or inappropriate projections of population growth [20]. As noted, fluctuation in Ethiopia’s official estimates of the denominator appear to account for little of the large year-to-year fluctuations seen in administrative coverage estimates.

Review of WHO data for 173 countries suggests that administrative data yield substantial (≥ 20 percentage points) over-estimates of coverage for at least one third of lower coverage countries in the world, including 5 of the 10 countries with the highest number of under-immunized children (see Additional file 3). Similar results were obtained for 103 countries from comparison of administrative estimates with the findings from their most recent nationally representative household survey (see Additional file 3).

Given the shortcomings of administrative estimates, it is important to periodically compare them with findings from population-based surveys. Some have argued that “DHS and MICS will usually be sufficient to monitor trends” [21]. Others emphasize the continued need to supplement DHS and MICS (UNICEF’s Multiple Indicator Cluster) surveys with EPI surveys [22]. Coverage surveys have their own limitations, including both “sampling error”, which can be reduced by increasing the sample size, and “non-sampling error” due to less rigorous sampling and data collection methods, recall bias and/or inaccurate records [3, 21, 23–25]. Sampling error can be estimated and is routinely noted in DHS and MICS reports for key indicators. The direction and magnitude of non-sampling error, however, can seldom be measured [23].

Most of the classic WHO EPI cluster surveys have been conducted with non-probability sampling, and may have been affected by selection bias and their reports frequently failed to adequately describe the methods used [2, 3, 23]. WHO’s latest guidelines now call for more rigorous sampling methods [17, 26] such as those used with Ethiopia’s 2012 EPI Survey. Higher quality surveys (including DHS and MICS, but also higher quality EPI surveys) invest considerable resources and technical oversight to overcome such selection bias and increase the sample size. As a result, they are typically expensive and several years elapse between them [22]. The cost for the 2012 EPI Survey appears relatively higher than either of the previous two EPI surveys as a result of the probability sampling methodology, closer quality control and additional field time required to seek documentation at nearby health facilities. (Personal communication, Abebe Bekele, EPHI).

As noted, compared to Ethiopia’s DHS surveys, each of the nationwide EPI surveys has had a larger sample size and a higher percentage of immunization data based upon written documentation. The authors cannot say to what extent such findings can be generalized to the surveys conducted in other countries. Given what is known about the methodological weaknesses common to many EPI surveys, the closer concordance between administrative estimates, WUENIC estimates and EPI survey estimates should not be taken as robust evidence that the EPI survey estimates are more reliable than DHS survey estimates. Nonetheless, EPI focal persons cited such findings when asked why they preferred estimates derived from EPI surveys.

Even with the largest of sample sizes and most rigorous of sampling and data collection methods, immunization coverage surveys can be affected by recall error when assessment is not based on data recorded on an HBR or clinic register. Such error may increase as immunization schedules become more complex with multiple vaccines administered at the same clinic visit and as questionnaires for the DHS and MICS grow in length. A review of 101 DHS and MICS surveys conducted from 1990 to 2000 found that vaccination data were obtained from recall for 45% of children due to non-availability of HBRs [27]. Ethiopia’s 2011 DHS found that only 65% of children had ever been issued an HBR and 56% of HBR’s ever issued had been lost [1]. The experience of the 2012 EPI survey demonstrates how difficult it can be to obtain documentation of vaccination status. Mothers were notified in advance of the survey and asked to have their vaccination cards ready. Despite this and facility trace-back exercises, only 47% of children in the 2012 EPI survey had a card available and only another 16% of children had vaccination status verifiable from the facility EPI register. The vaccination status of the remaining 37% of children therefore had to be based upon history [10].

Studies in low to middle income countries have found that recall error resulted in over-estimation of immunization coverage (up to 43 percentage points) in some populations [28–32], and under-estimation (up to 10 percentage points) in others [25, 33–37].

It should be noted that WUENIC estimators adjust some DPT3 estimates to partially address recall error: “Whenever estimates are based primarily on survey data and the proportion of vaccinations based on maternal recall is high, survey coverage levels are adjusted to compensate for maternal recall for multi-dose antigens (i.e. DPT, polio vaccine, …) by applying the dropout between the first and third doses observed in the documented data to the vaccination history reported by the child’s caretaker*.”* [6] The same adjustment was reported for the 2012 EPI survey, although the unadjusted finding is shown in Fig. 1 [10].

Our secondary analysis of data from the 2012 Ethiopian Immunization Coverage Survey showed that recall under-estimated documented DPT3 coverage by 30 percentage points for those children for which documentation could be found at a health facility. It should not be assumed that the children for whom facility records could be relocated is representative of all children for whom HBR’s are unavailable. Children for whom no facility register could be found are more likely to have not been immunized. Nonetheless, these findings suggest that recall bias can result in substantial under-estimation of immunization coverage in Ethiopia. This is consistent with findings from secondary analysis of data from 3 woredas in Ethiopia compiled by Travossos et al [38] showing that DPT 3 coverage based on caretaker recall was 10 percentage points lower than coverage documented for the same children on clinics registers and 27 percentage points lower than the prevalence of protective anti-tetanus antibodies in these children.

The consecutive surveys shown in Fig. 1 provide coverage estimates for different years. However, the substantial differences between the survey estimates of consecutive years without significant differences in the administrative estimates of those years, suggest that there are important differences in non-sampling errors between the different types of surveys.

Nigeria provides another example where strikingly different estimates of immunization coverage were obtained from two robust household surveys, conducted less than 2 years apart, each with a standard error of less than 2 percentage points: DPT3 coverage was measured as 34% for the 2016/2017 MICS versus 57% for the 2018 DHS. For both surveys, immunization status was documented with home-based records for less than 40% of children. For the 2018 DHS, data from HBR’s was supplemented with data from facility-based records. A paper by Dong et al. [39] attributes much of the inconsistency between findings of different surveys in Nigeria to the state-level weights that were used.

WHO/UNICEF Estimates of National Immunization Coverage have all of the same limitations just described. Since 2012 (including for retrospective estimates going back to 2000) WUENIC reports have noted the “Grade of Confidence” (GoC) for each yearly WUENIC estimate [40]. A 3-star scale is used to indicate the extent to which each year’s WUENIC estimate is consistent with administrative and survey estimates. Not surprisingly, given the above findings, the GoC has been one star for all WUENIC estimates for Ethiopia – indicating that the WUENIC estimate conflicted with either the administrative estimate or the survey estimate or both by greater than 10 percentage points.

WHO’s latest guidelines [2] will hopefully help strengthen the statistical rigor of survey estimates without sharply reducing their frequency. For countries such as Ethiopia, one high-quality, nationally representative coverage survey each 5 years is not sufficient. The challenge will be to design and conduct future surveys in such a way that the results are fully accepted and used by health officials. Further research is warranted on how best to assure the comparability of surveys using different questionnaires or methodologies.

Most of the above cited limitations of the different methodologies were noted by the key informants interviewed as part of this study. EPI staff recognised the limitations of data routinely reported by health facilities. However, there was a general preference for administrative data, related less to their reliability than to their capacity to inform timely decision-making at the most decentralized level. It is possible that higher confidence in administrative data as opposed to surveys stems from interviewees’ desirability bias. However, this might also reflect the lack of familiarity with and understanding of survey methodology and a better alignment of administrative data with local programmatic incentives. Reluctance to make greater use of survey estimates might be reduced by limiting the length of survey questionnaires [41], by making greater use of small population-based health surveys (district level and below) [42] and by assuring greater involvement of health officials, including EPI managers, in survey planning, implementation and analysis [2, 17]. While we acknowledge that our study only surveyed staff from the immunization programme who may not be representative of other health officials, the sample of respondents was large and from a wide range of locations and administrative levels. It provides valuable understanding of how data is understood and used by end users and how this influences the framing of the FMoH Annual Performance Report.

In recent years, the Ministry of Health has pursued reforms on its health data systems as part of an “Information Revolution”. These reforms have focussed on the simplification, standardization and integration of the reporting and use of administrative data. According to the most recent Annual Performance report of the FMoH, there have been significant improvements in the availability and completeness of source documents and report accuracy [14]. .Here, the recommendations offered by the EPI focal persons who were interviewed for this study warrant further attention. In particular, greater emphasis should be placed upon routinely validating monthly reports with data from facility registers and the number of doses of vaccine supplied.

Given the history of discrepancies between administrative and survey estimates and the erratic year-to-year fluctuations in estimates from each of these sources, those interpreting these findings would do well to seek out data from additional sources (i.e. vaccine supply data, data verification surveys comparing administrative reports with data on facility registers, surveillance data). With triangulation of data from multiple sources, Ethiopia’s actual immunization coverage could be estimated with greater confidence.

## Conclusions

The findings from this case study underscore the need for global investments to improve country health data – administrative data, target estimates, and survey data. For now, estimates of immunization coverage, regardless of how they were derived, should be interpreted with caution. For many countries, especially those with coverage of 90% or more, estimates based upon administrative data have in recent years been consistent with estimates derived from surveys and other evidence. The discrepancies and the uncertainty are greater for the countries with lower coverage which account for the great majority of the under-vaccinated children in the world.

It is important that the focus on the reliability of national estimates not obscure our recognition that reliable routine data are essential for local decision makers -- to identify under-vaccinated children and manage the interventions needed to reach them. Where there are substantial discrepancies between routine and survey estimates of coverage, the response must not be to dismiss one estimate or another. Rather, it is essential to identify and address the reasons for such discrepancy and improve the *acceptability* of findings from each data source.

## Supplementary Information

**Additional file 1.** Text of questionnaire followed by table showing the number of informants by geographic region.

**Additional file 2.** A chart comparing the 20-year trend in official numerator data to the 20-year trend in the official estimate of the denominator, as reported to WHO/UNICEF on the annual Joint Reporting Form [5].

**Additional file 3.** The first table summarizes findings for 103 countries from comparison of DPT3 coverage as measured by the most recent population-based survey conducted in the last 10 years versus the administrative estimate for children of the same birth cohort. The second table summarizes findings from comparison of the 2018 administrative versus WUENIC estimates of national DPT3 coverage for the 173 countries which reported their administrative data to WHO and UNICEF.

## Data Availability

The datasets used and analysed during the current study are available from the corresponding author. An online repository has been set up at https://drive.google.com/drive/folders/1at0kuppEM3kmymDdtrQzV7K5HOEUBWw4?usp=sharing to access copies of official reports which readers may find difficult to access from other addresses on the world wide web.

## Abbreviations

DHS: Demographic and Health Survey
DPT 3: Third dose of the Diptheria-Pertussis-Tetanus or Penta vaccine
EPI: Expanded Programme for Immunization
FMoH: Federal Ministry of Health of Ethiopia
HMIS: Health Management Information System
JRF: The Joint Reporting Form of WHO and UNICEf
MICS: Multiple Indicator Cluster Survey
NIP: National Immunization Programme
UN: United Nations
WHO: World Health Organisation
WUENIC: WHO/UNICEF estimates of national immunization coverage
