# Supplementary material for: Estimation of Ethiopia’s immunization coverage – 20 years of discrepancies
Source: BMC Health Serv Res. 2021 Sep 13;21(Suppl 1):587. doi: 10.1186/s12913-021-06568-0 (PMC8436460; doi:10.1186/s12913-021-06568-0)
Supplement: Supplementary file 1 — Additional file 1. Text of questionnaire followed by table showing the number of informants by geographic region. [file 12913_2021_6568_MOESM1_ESM.docx]

**Questionnaire and geographic distribution of interviews**

**with health officials involved with management of the immunization programme**

**Questionnaire**

**Introductory statement**: Often there are large differences between the vaccination coverage estimates obtained from different data sources. For example, for 2010 and 2011 the DPT3 coverage estimate based upon HMIS data was 86%. However, the estimate from the 2011 DHS was 37% and the estimate from the 2012 EPI survey was 60%. There is a wide gap.

- Question 1: What could be the main reasons for the large differences between these estimates?
- Question 2: In your opinion which data source is more reliable and why? (EPI coverage survey, EDHS or HMIS)
- Question 3: Which data source is sometimes unreliable and why? (EPI coverage survey, EDHS or HMIS
- Question 4: What should be done to improve the reliability of coverage estimates?

**Geographic distribution of interviews**

| **Region / City** | **Zones** | **Local health officials**  **interviewed at zonal level** | | **Local health officials**  **interviewed at regional level** | **Total health officials**  **interviewed** |
| --- | --- | --- | --- | --- | --- |
| Tigray | 6 | 6 | | 1 | 7 |
| Afar | 5 | 5 | | 1 | 6 |
| Amhara | 13 | 13 | | 1 | 14 |
| Oromia | 24 | 24 | | 1 | 25 |
| Somali | 10 | 10 | | 1 | 11 |
| Benishangul Gumuz | 4 | 4 | | 1 | 5 |
| Gambella | 4 | 4 | | 1 | 5 |
| SNNPR | 19 | 19 | | 1 | 20 |
| Hareri | 2 | 0 | | 1 | 1 |
| Dire Dawa | 2 | 0 | | 1 | 1 |
| Addis Ababa | 10 | 10 | | 1 | 11 |
| Nationwide | 99 | | 95 | 11 | 106 |
