# Supplementary material for: Estimation of Ethiopia’s immunization coverage – 20 years of discrepancies
Source: BMC Health Serv Res. 2021 Sep 13;21(Suppl 1):587. doi: 10.1186/s12913-021-06568-0 (PMC8436460; doi:10.1186/s12913-021-06568-0)
Supplement: Supplementary file 2 — Additional file 2. A chart comparing the 20-year trend in official numerator data to the 20-year trend in the official estimate of the denominator, as reported to WHO/UNICEF on the annual Joint Reporting Form [5]. [file 12913_2021_6568_MOESM2_ESM.docx]

**Reported DPT 3rd doses and official estimates of surviving infants, Ethiopia, 2000 to 2019 -- as reported on the Joint Reporting Form** [5]

**Interpretation**: While the denominator has not always risen at a constant rate, most of the erratic year-to-year fluctuations and longer-term rise in the administrative estimate of DPT3 coverage has been due to marked instability and upwards drift in the numerator.
