# Supplementary material for: Estimation of Ethiopia’s immunization coverage – 20 years of discrepancies
Source: BMC Health Serv Res. 2021 Sep 13;21(Suppl 1):587. doi: 10.1186/s12913-021-06568-0 (PMC8436460; doi:10.1186/s12913-021-06568-0)
Supplement: Supplementary file 3 — Additional file 3. The first table summarizes findings for 103 countries from comparison of DPT3 coverage as measured by the most recent population-based survey conducted in the last 10 years versus the administrative estimate for children of the same birth cohort. The second table summarizes findings from comparison of the 2018 administrative versus WUENIC estimates of national DPT3 coverage for the 173 countries which reported their administrative data to WHO and UNICEF. [file 12913_2021_6568_MOESM3_ESM.docx]

**How common are discrepancies in immunization coverage estimates?**

Table 1 summarizes findings for 103 countries from comparison of DPT3 coverage as measured by the most recent population-based survey conducted in the last 10 years versus the administrative estimate for children of the same birth cohort [5]. For 24 (71%) of 34 countries with lower coverage (retrospective WUENIC DPT3 estimate < 85% for the year assessed by the survey), the administrative estimate was more than 10 percentage points higher than the survey estimate. For 8 of the 10 countries which, according to their 2018 WUENIC estimates, had the largest numbers of under-vaccinated children, the administrative estimates were more than 10 percentage points higher than their most recent survey estimates. According to the UN estimates, together these 8 countries accounted for 43% of all under-vaccinated children in the world in 2018. In contrast, for 48 (70%) of the 69 higher coverage countries, the contemporaneous administrative estimate was within 10 percentage points of the survey estimate.

Table 1: Difference between the survey estimate of DPT3 coverage and the administrative estimate for the same birth cohort, most recent national survey since 2000

| **Administrative estimate minus survey estimate** | **WUENIC* DPT3 for year assessed by the survey** | | **The 10 countries with the largest number of under-vaccinated infants in 2018** |
| --- | --- | --- | --- |
|  | < 85% | ≥ 85% |  |
| ≥40 percentage points | 5/34 (14%) | 0/69 | Angola, Nigeria, Chad |
| ≥30 to >40 percentage points | 5/34 (14%) | 0/69 | Ethiopia, DR Congo, Indonesia |
| ≥20 to >30 percentage points | 8/34 (24%) | 4/69 (6%) | Afghanistan |
| ≥10 to >20 percentage points | 6/34 (18%) | 10/69 (14%) | Pakistan |
| Within 10 percentage points | 9/34 (26%) | 48/69 (70%) | India, Philippines |
| ≤ negative 10 percentage points | 1/34 (3%) | 7/69 (10%) |  |

* Retrospective WUENIC estimate published in 2019.

Table 2 summarizes findings from comparison of the 2018 administrative estimates of national DPT3 coverage for the 173 countries which reported their administrative data to WHO and UNICEF^5^. For more than half (55%) of the countries with lower coverage (2018 WUENIC DPT3 estimate < 85%), and for 8 of the 10 countries with the largest number of under-vaccinated children (accounting for 56% of the under-vaccinated children in the world, according to 2018 WUENIC estimates), the administrative estimate was more than 10 percentage points higher than the WUENIC estimate. In contrast, for 90% of the higher coverage countries, the administrative estimate was within 10 percentage points of the WUENIC estimate.

Table 2: Difference between 2018 administrative estimate and 2018 WUENIC estimate of DPT3 coverage, 173 countries reporting their administrative data to WHO and UNICEF

| **Administrative estimate minus WUENIC estimate** | **2018 WUENIC estimate** | | **The 10 countries with the largest number of under-vaccinated infants in 2018** |
| --- | --- | --- | --- |
|  | < 85% | ≥ 85% |  |
| ≥40 percentage points | 1/47 (2%) | 0/126 (0%) |  |
| ≥30 to >40 percentage points | 4/47 (9%) | 0/126 (0%) | Chad, Nigeria |
| ≥20 to >30 percentage points | 8/47 (17%) | 0/126 (0%) | Afghanistan, Angola, Ethiopia |
| ≥10 to >20 percentage points | 13/47 (28%) | 4/126 (3%) | DR Congo, India, Indonesia |
| Within 10 percentage points | 20/47 (43%) | 113/126 (90%) | Pakistan, Philippines |
| ≤ negative10 percentage points | 1/47 (2%) | 9/126 (7%) |  |
